# Is Large Language Model-Assisted Citation Screening Feasible in a Scoping Review on Nonpharmacological Interventions for Delirium in Patients With Cancer?

**DOI:** 10.7759/cureus.90026

**Published:** 2025-08-13

**Authors:** Yoshiyasu Ito, Hironobu Ikehara, Yoshiaki Okamoto, Jun Kako

**Affiliations:** 1 Faculty of Nursing Science, Tsuruga Nursing University, Tsuruga, JPN; 2 Department of Nursing, Graduate School of Medicine, Mie University, Tsu, JPN

**Keywords:** artificial intelligence (ai), cancer, large language models, nonpharmacological intervention, scoping review

## Abstract

Systematic and scoping reviews are essential in palliative care, yet they are time-consuming and resource-intensive. Recent advancements in artificial intelligence, particularly large language models (LLMs), have shown promise in enhancing the efficiency of literature screening. However, their feasibility and accuracy in scoping reviews remain unclear. In this study, we aimed to evaluate the feasibility and performance of LLM-assisted citation screening for a scoping review on nonpharmacological interventions for delirium in patients with cancer. This prospective simulation study assessed the accuracy of three LLMs, GPT-4 Turbo, GPT-4o, and model o1 (OpenAI, San Francisco, CA, USA), in screening titles and abstracts. The dataset was derived from a previously conducted scoping review. Two reference standards were used for comparison: title/abstract screening and full-text screening results from conventional human review. LLMs were prompted using standardized inclusion and exclusion criteria based on the Population, Concept, and Context (PCC) framework. Sensitivity, specificity, positive predictive value (PPV), and negative predictive value (NPV) were calculated for each model. The sensitivity and specificity were 0.43 (95% CI, 0.06-0.80) and 0.99 (95% CI, 0.99-1.00) for GPT-4 Turbo, 0.71 (95% CI, 0.38-1.00) and 0.97 (95% CI, 0.96-0.98) for GPT-4o, and 1.00 (95% CI, 1.00-1.00) and 0.91 (95% CI, 0.89-0.92) for o1, respectively. Compared with reference standard 2 (full-text screening results from conventional citation screening), the sensitivity and specificity were 1.00 (95% CI, 1.00-1.00) and 0.99 (95% CI, 0.99-1.00) for GPT-4 Turbo, 1.00 (95% CI, 1.00-1.00) and 0.97 (95% CI, 0.96-0.98) for GPT-4o, and 1.00 (95% CI, 1.00-1.00) and 0.90 (95% CI, 0.89-0.92) for o1, respectively. All models demonstrated high NPVs, indicating strong reliability in excluding irrelevant studies. However, PPVs were low across all models, reflecting a high false-positive rate. Newer LLMs, particularly model o1, demonstrated high sensitivity and acceptable specificity, supporting their use as preliminary screening tools in scoping reviews. High NPVs suggest LLMs are reliable for ruling out irrelevant citations, thereby streamlining the initial screening phase. However, consistently low PPVs raise concerns about increased reviewer burden due to false positives, emphasizing the necessity of human validation. These findings support the cautious integration of LLMs into literature screening workflows, treating their outputs as supportive tools rather than replacements for expert judgment.

## Introduction

Systematic reviews (SRs) integrating the latest evidence are essential for clinical decision-making in palliative care. However, SRs require substantial time and human resources, which artificial intelligence technologies can overcome, particularly large language models (LLMs) [1-3]. LLMs are a category of foundation models trained on immense amounts of data, making them capable of understanding and generating natural language and other types of content to perform a wide range of tasks [4].

Using specific selection criteria, LLMs can effectively screen the literature, demonstrating high sensitivity and specificity for inclusion and exclusion decisions [5]. Hence, LLM-assisted screening is anticipated to enhance SR efficiency and reduce workload.

Before conducting SRs, scoping reviews are often performed to map the extent and type of available evidence [6], again requiring considerable time and human resources due to reliance on rigorous literature screening. Consequently, LLM-assisted screening could also be beneficial for scoping reviews; however, the feasibility of this approach is unclear. Herein, we evaluated the feasibility and accuracy of LLM-assisted citation screening in a scoping review process.

## Technical report

Technical implementation of LLM-assisted citation screening

In this prospective simulation study, we evaluated the accuracy of three LLMs in screening citation titles and abstracts. Data were obtained from a conventional scoping review process on nonpharmacological interventions for delirium in patients with cancer [7]. Nonpharmacological interventions for delirium in patients with cancer were defined as non-drug approaches used for the prevention or treatment of delirium in patients diagnosed with any type of cancer. Herein, literature selected by two independent reviewers was used as a reference standard. Screening was conducted from April 27, 2025.

Three LLMs, GPT-4 Turbo, GPT-4o, and o1 (OpenAI, San Francisco, CA, USA), were used to independently conduct LLM-assisted citation screening. The program enabling these LLMs to autonomously perform citation screening was developed and executed using Python (version 3.12; Python Software Foundation, Fredericksburg, VA, USA) and Pandas (version 2.2.2; NumFOCUS, Inc., Austin, TX, USA), implemented via an application programming interface, following a previously established methodology [5]. The prompt used to instruct the LLMs in screening literature according to inclusion and exclusion criteria was adapted from a previously established methodology [5]. This prompt was designed to guide the LLMs in performing citation screening based on the inclusion and exclusion criteria defined in the conventional screening process. Specifically, it instructed the LLMs to apply criteria based on the Population, Concept, and Context (PCC) framework and study design. The LLMs were instructed to assess the title and abstract of each study according to predefined eligibility criteria-specifically, (1) population: patients with cancer and delirium (≥80% cancer population), (2) concept: nonpharmacological interventions for delirium, (3) context: all settings, and (4) study design: all designs. Studies focusing on sedation, causative treatments, or written in languages other than English or Japanese were excluded. In cases of uncertainty due to insufficient information, the model was instructed to err on the side of inclusion. The programming code and full version of the prompts used in this study are available on the GitHub repository [8].

Methods for evaluating screening performance

To evaluate the accuracy of LLM-assisted citation screening, we compared the screening results produced by GPT-4 Turbo, GPT-4o, and o1 with two reference standards. Reference standard 1 was defined as the title and abstract screening results obtained through conventional citation screening. In contrast, reference standard 2 was defined as the full-text screening results obtained through conventional citation screening. For each comparison, sensitivity, specificity, positive predictive value (PPV), and negative predictive value (NPV) were calculated.

Screening performance of LLM-assisted citation methods

The results of the analysis are summarized in Table 1. When compared with reference standard 1 (title and abstract screening results from conventional citation screening), the sensitivity and specificity were 0.43 (95% CI, 0.06-0.80) and 0.99 (95% CI, 0.99-1.00) for GPT-4 Turbo, 0.71 (95% CI, 0.38-1.00) and 0.97 (95% CI, 0.96-0.98) for GPT-4o, and 1.00 (95% CI, 1.00-1.00) and 0.91 (95% CI, 0.89-0.92) for o1, respectively. In comparison with reference standard 2 (full-text screening results from conventional citation screening), the sensitivity and specificity were 1.00 (95% CI, 1.00-1.00) and 0.99 (95% CI, 0.99-1.00) for GPT-4 Turbo, 1.00 (95% CI, 1.00-1.00) and 0.97 (95% CI, 0.96-0.98) for GPT-4o, and 1.00 (95% CI, 1.00-1.00) and 0.90 (95% CI, 0.89-0.92) for o1, respectively. The complete dataset, including the screening outcomes for each reference, is publicly available through a dedicated GitHub repository [8]. The citation screening and analytical process is presented in the Appendices.

**Table 1:** Sensitivity and specificity of large language model-assisted citation screening CI, confidence interval; NPV, negative predictive value; PPV, positive predictive value. ^a ^Using title and abstract screening results as the reference standard. ^b ^Using full-text screening results as the reference standard.

Accuracy metrics	GPT-4-turbo	GPT-4o	o1
Reference standard 1 ^a^			
Sensitivity (95% CI)	0.43 (0.06-0.80)	0.71 (0.38-1.00)	1.00 (1.00-1.00)
Specificity (95% CI)	0.99 (0.99-1.00)	0.97 (0.96-0.98)	0.91 (0.89-0.92)
PPV (95% CI)	0.23 (0.00-0.46)	0.11 (0.02-0.20)	0.05 (0.02-0.09)
NPV (95% CI)	1.00 (0.99-1.00)	1.00 (1.00-1.00)	1.00 (1.00-1.00)
Reference standard 2 ^b^			
Sensitivity (95% CI)	1.00 (1.00-1.00)	1.00 (1.00-1.00)	1.00 (1.00-1.00)
Specificity (95% CI)	0.99 (0.99-1.00)	0.97 (0.96-0.98)	0.90 (0.89-0.92)
PPV (95% CI)	0.08 (0.00-0.22)	0.02 (0.00-0.07)	0.01 (0.00-0.02)
NPV (95% CI)	1.00 (1.00-1.00)	1.00 (1.00-1.00)	1.00 (1.00-1.00)

## Discussion

This study evaluated the feasibility of using LLMs for citation screening in a scoping review on nonpharmacological interventions for delirium in cancer patients. Newer models, such as OpenAI’s o1, which has demonstrated enhanced reasoning capabilities in medical contexts [9], achieved higher sensitivity, matching human performance at both the abstract and full-text levels. While GPT-4 Turbo showed extremely high specificity, its low sensitivity at the abstract level suggests a conservative approach, likely excluding studies when eligibility is not explicitly stated [10]. GPT-4o and o1 demonstrated more balanced performance, maintaining high specificity while achieving greater sensitivity even when abstracts lacked clear PCC criteria. This suggests that newer models like model o1 can interpret implicit contextual cues better. In addition, all LLMs demonstrated consistently high NPVs, indicating that studies excluded by the models were indeed unlikely to be relevant. Consistently high NPVs have also been reported in previous studies, highlighting this as a particular strength of LLMs [11-13]. This suggests that LLMs may be reliable tools for eliminating irrelevant citations, thereby streamlining the initial phase of literature screening. Prior research has indicated that the effectiveness of LLM-assisted screening is highly dependent on the specific model employed, with outcomes varying substantially across different LLMs [1,14]. Nonetheless, the results of our study suggest that newer models, such as o1, may function as effective tools for first-pass screening in the early stages of scoping reviews, thereby contributing to a reduction in the overall screening workload.

However, PPVs were notably low across all models, indicating that a substantial proportion of studies identified as potentially relevant by the LLMs were ultimately deemed ineligible upon further review. The issue of low PPV has also been consistently highlighted in previous studies [13,15]. In addition, LLMs can exhibit reference hallucinations [16], in which they generate citations that are nonexistent, inaccurate, or irrelevant. This phenomenon is believed to be caused by several factors, including potential defects in the transformer-based encoding and decoding processes within the model [16]. Without careful verification, such hallucinations may further exacerbate the false-positive rate. This raises a critical concern that, rather than reducing human workload, LLM-assisted inclusion may inadvertently increase the burden of review by introducing large volumes of false positives. In practice, this could lead to inefficient allocation of reviewer time and resources, particularly if LLM outputs are interpreted as high-confidence recommendations rather than preliminary filters. These findings underscore the need for careful integration of LLMs into screening workflows, with human validation remaining essential, in particular, for inclusion decisions. As a practical strategy to mitigate this risk, LLM outputs should be used solely for preliminary screening, followed by a two-stage screening process in which independent human reviewers verify all inclusion decisions.

This study had some limitations. First, the study was based on a single scoping review topic, which may limit generalizability to other clinical areas with different terminology, complexity, or volume of evidence. Second, although two independent reference standards were used, both were derived from a conventional human screening process and may contain subjective variability or undetected errors. Third, while the prompts were adapted from previous literature, their optimization for specific research domains remains an open challenge. Finally, the simulation setting does not fully capture the iterative nature of real-world citation screening, where feedback loops and team discussions play a crucial role. These limitations underscore the importance of cautious interpretation and call for further validation studies across diverse review topics and LLM configurations.

## Conclusions

LLMs such as GPT-4 Turbo, GPT-4o, and o1, particularly newer versions, may serve as effective tools for first-pass screening, potentially reducing workload in the early stages of scoping reviews. However, the low PPVs across all models indicate that many studies flagged as relevant by LLMs were ultimately ineligible. This could increase reviewer workload due to the large number of false positives; in large-scale review processes or fully automated workflows, such limitations could result in substantial inefficiencies. These findings highlight the importance of treating LLM outputs as preliminary filters rather than final decisions, with human validation remaining essential.

## Figures and Tables

**Figure 1:**
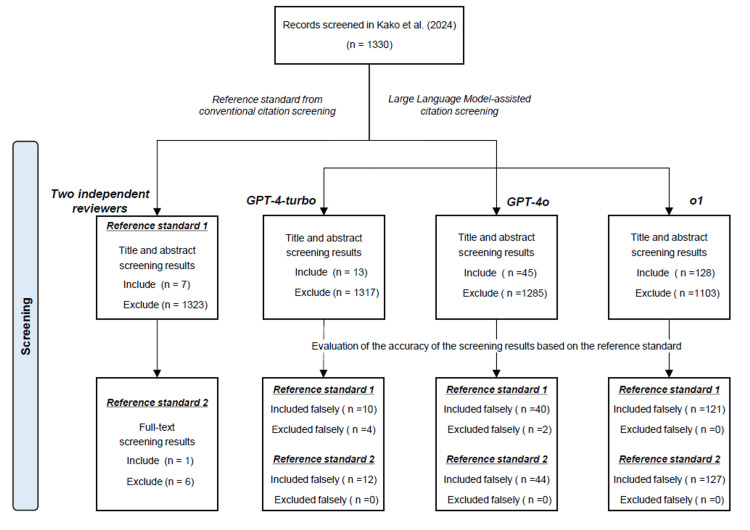
Large language model-assisted citation screening and analytical process Based on: [7]
